# Neuronal ceroid lipofuscinosis type 2: an Australian case series

**DOI:** 10.1111/jpc.14890

**Published:** 2020-04-24

**Authors:** Alexandra M Johnson, Simone Mandelstam, Ian Andrews, Katja Boysen, Joy Yaplito‐Lee, Michael Fietz, Lakshmi Nagarajan, Victoria Rodriguez‐Casero, Monique M Ryan, Nicholas Smith, Ingrid E Scheffer, Carolyn Ellaway

**Affiliations:** ^1^ Department of Neurology Sydney Children's Hospital Sydney New South Wales Australia; ^2^ Department of Paediatrics University of Melbourne Melbourne Victoria Australia; ^3^ Department of Radiology University of Melbourne Melbourne Victoria Australia; ^4^ Imaging and Epilepsy Group The Florey Institute of Neuroscience and Mental Health Melbourne Victoria Australia; ^5^ Department of Paediatric Radiology The Royal Children's Hospital Melbourne Melbourne Victoria Australia; ^6^ Murdoch Children's Research Institute The Royal Children's Hospital Melbourne Melbourne Victoria Australia; ^7^ Department of Paediatrics The Royal Children's Hospital Melbourne Melbourne Victoria Australia; ^8^ Department of Metabolic medicine The Royal Children's Hospital Melbourne Melbourne Victoria Australia; ^9^ Clinical Informatics Illumina Australia Melbourne Victoria Australia; ^10^ Diagnostic genomics PathWest Laboratory Medicine WA Perth Western Australia Australia; ^11^ National Referral Laboratory SA Pathology Adelaide South Australia Australia; ^12^ Children's Neuroscience Service Perth Children's Hospital Perth Western Australia Australia; ^13^ Faculty of Health and Medical Sciences The University of Western Australia Perth Western Australia Australia; ^14^ Neurology Department The Royal Children's Hospital Melbourne Melbourne Victoria Australia; ^15^ Department of Neurology and Clinical Neurophysiology Women's and Children's Hospital Adelaide South Australia Australia; ^16^ Adelaide Medical School The University of Adelaide Adelaide South Australia Australia; ^17^ Department of Neurology Austin Health Melbourne Victoria Australia; ^18^ Genetic Metabolic Disorders Service The Sydney Children's Hospitals Network Sydney New South Wales Australia; ^19^ Disciplines of Genetic Medicine and Child and Adolescent Health The University of Sydney Sydney New South Wales Australia

**Keywords:** cerebellar atrophy, cerebral atrophy, ceroid lipofuscinosis type 2 disease, epilepsy, language delay, magnetic resonance imaging

## Abstract

**Aim:**

Late infantile neuronal ceroid lipofuscinosis type 2 (CLN2) disease is a rare neurodegenerative disorder presenting in children aged 2–4 years with seizures and loss of motor and language skills, followed by blindness and death in late childhood. Initial presenting features are similar to a range of common epilepsies. We aim to highlight typical clinical and radiological features that may prompt diagnosis of CLN2 disease in early disease stages.

**Methods:**

We present a series of 13 Australian patients with CLN2 disease, describing clinical features, disease evolution, neuroimaging, electroencephalogram, biochemical and genetic results. Expert neuroradiological magnetic resonance imaging (MRI) analysis was retrospectively performed on 10 cases.

**Results:**

Twelve patients presented with seizures, with initial seizures being focal (*n* = 4), generalised tonic–clonic (*n* = 3), absence (*n* = 3) and febrile (*n* = 2). Eleven patients (85%) had a language delay before the onset of seizures. Cerebellar or cerebral atrophy was noted in all patients on centralised MRI review, with abnormalities of the brain‐stem, ventricles, corpus callosum and hippocampi.

**Conclusions:**

Early language delay with the onset of seizures at 2–4 years of age is the hallmark of CLN2 disease. MRI findings of early subtle atrophy in the cerebellum or posterior cortical regions should hasten testing for CLN2 disease to enable early initiation of enzyme replacement therapy.

## What is already known on this topic

1. Ceroid lipofuscinosis type 2 (CLN2) disease is a rare, rapidly progressive paediatric disease, presenting with early language delay and seizures in children 2–4 years
2. Diagnostic delays are common due to its rarity and non‐specific presentation
3. Early diagnosis is key to ensure appropriate disease‐specific management strategies are in place prior to disease progression

## What this paper adds

1. Highlights need for intermittent photic stimulation to be included with all electroencephalogram in this age group to assist with early diagnosis of CLN2 disease
2. Highlights pattern of magnetic resonance imaging changes in CLN2 disease with cerebellar and cerebral atrophy and changes in white matter, brain‐stem and hippocampus
3. Improved delineation of presenting epilepsy semiology in CLN2 disease

The neuronal ceroid lipofuscinoses (NCLs) are a heterogeneous group of lysosomal storage disorders and are the most common cause of childhood dementia.^1^ NCLs are characterised by intra‐lysosomal accumulation of autofluorescent storage material (ceroid lipofuscin) in neurons and other tissues, which results in neuronal death.^2^, ^3^, ^4^ CLN2 disease is one of the most common NCLs, caused by deficiency of the tripeptidyl peptidase 1 (TPP1) enzyme secondary to mutations in the *CLN2* gene.^2^, ^3^, ^5^, ^6^ The estimated incidence is <0.5 per 100 000 live births.^5^, ^6^ Recent figures indicate a similar incidence of 0.77 per 100 000 live births in Australia based on diagnoses through lysosomal enzyme testing at national testing centres (personal communication) and the Australian birth rate.

CLN2 disease is characterised by early language delay, seizures and ataxia, with epilepsy beginning between ages 2 and 4 years.^1^, ^2^, ^3^, ^6^, ^7^, ^8^, ^9^ Language delay often precedes the onset of seizures.^6^, ^7^ Multiple seizure types often occur, including generalised tonic–clonic, myoclonic, absence and focal seizures.^7^ These seizures are often refractory to antiepileptic medications.^1^, ^6^ Disease progression is rapid over 2–3 years, leading to regression of developmental skills, movement disorders, dementia and visual loss,^1^, ^2^, ^3^ Late‐stage disease is typically reached by 6 years, and can last for several years.^2^, ^6^, ^7^ Children become completely dependent upon their caregivers due to immobility and regression. They experience feeding difficulties, behavioural and sleep disturbances, and ongoing seizures, and are at risk of respiratory infection secondary to lack of mobility and poor ability to clear their airways. Patients usually die in late childhood or early adolescence, with a median age of death of 10 years in the DEM‐CHILD natural history registry.^2^, ^6^, ^8^

Diagnostic clues to CLN2 disease can include electroencephalogram (EEG) findings, particularly in response to intermittent photic stimulation (IPS) at slow flash frequencies of 1–3 Hz, and magnetic resonance imaging (MRI) patterns of atrophy. Characteristic findings on EEG may suggest the diagnosis of CLN2 disease, including the hallmark photoparoxysmal response (PPR) associated with low‐frequency (1–3 Hz) IPS.^10^, ^11^, ^12^ IPS can produce a PPR at low frequencies which has a high specificity for this disorder,^10^ but may not be routinely undertaken in all neurophysiology labs. Characteristic changes on brain MRI may also suggest CLN2 disease, with progressive cerebral and cerebellar atrophy, and periventricular white matter changes.^10^

Unlike other NCLs, CLN2 disease infrequently presents with visual impairment.^13^ Visual decline is common with disease progression.^2^, ^6^, ^9^, ^14^

**Figure 1:**
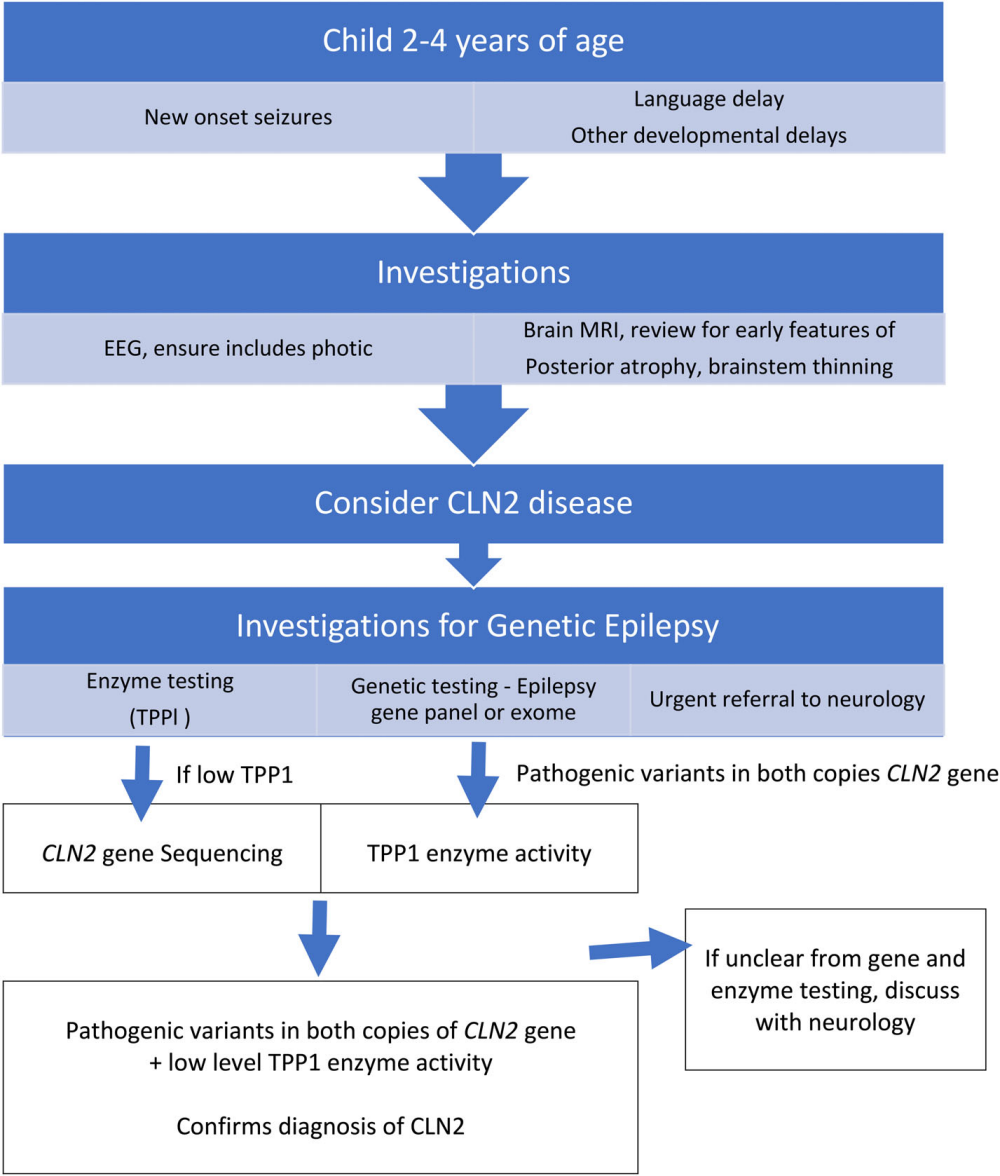
Path to diagnosis for CLN2 disease is through a combination of enzymatic and genetic investigations after initial suspicion is raised. CLN2, ceroid lipofuscinosis type 2; EEG, electroencephalogram; MRI, magnetic resonance imaging; TPP1, tripeptidyl peptidase 1.

Diagnosis of CLN2 disease is based on a combination of enzyme and gene testing (see Fig. 1 for suggested pathway to diagnosis). Diagnosis of CLN2 disease has been frequently delayed due to the rarity of the disease and its relatively non‐specific presenting features, together with limited awareness among paediatricians and paediatric neurologists and lack of access to diagnostic testing.^15^ Nickel *et al*. reported a mean delay of 22.7 months from seizure onset to diagnosis.^8^ However, early diagnosis is critical in terms of family planning, supportive therapies, disease‐specific management and potential access to clinical trials and novel therapies.^6^ Currently, there is no cure for CLN2 disease; until recently, management has focused on symptomatic, supportive and palliative treatment involving a multidisciplinary health‐care team.^6^ In 2017, enzyme replacement therapy (ERT) with cerliponase alfa was granted regulatory approval in the United States and Europe, providing the first disease‐specific treatment for patients with CLN2 disease and considerably improving the disease course.^16^ Cerliponase alfa was approved in 2019 in Australia and is available under the federal government Life Saving Drugs Program.

There is limited literature on the heterogeneous presentation and MRI features of patients with CLN2 disease. We, therefore, aim to delineate the clinical features, natural history and MRI features in a series of Australian patients to facilitate early diagnosis of CLN2 disease in early disease stages.

## Methods

This study was a retrospective review of a cohort of Australian patients diagnosed with CLN2 disease between 2004 and 2017. Cases were obtained from five centres from New South Wales, Victoria, South Australia and Western Australia. Informed consent was provided by the parents or legal guardians of each patient.

Data reviewed from medical files included clinical, neuroradiological and electrophysiological features, and molecular and metabolic investigation results. In addition, brain MRIs of 10 patients were reanalysed by an expert paediatric neuroradiologist (Simone Mandelstam). Scans from three patients were unavailable. Descriptive statistics are presented.

## Results

**Figure 2:**
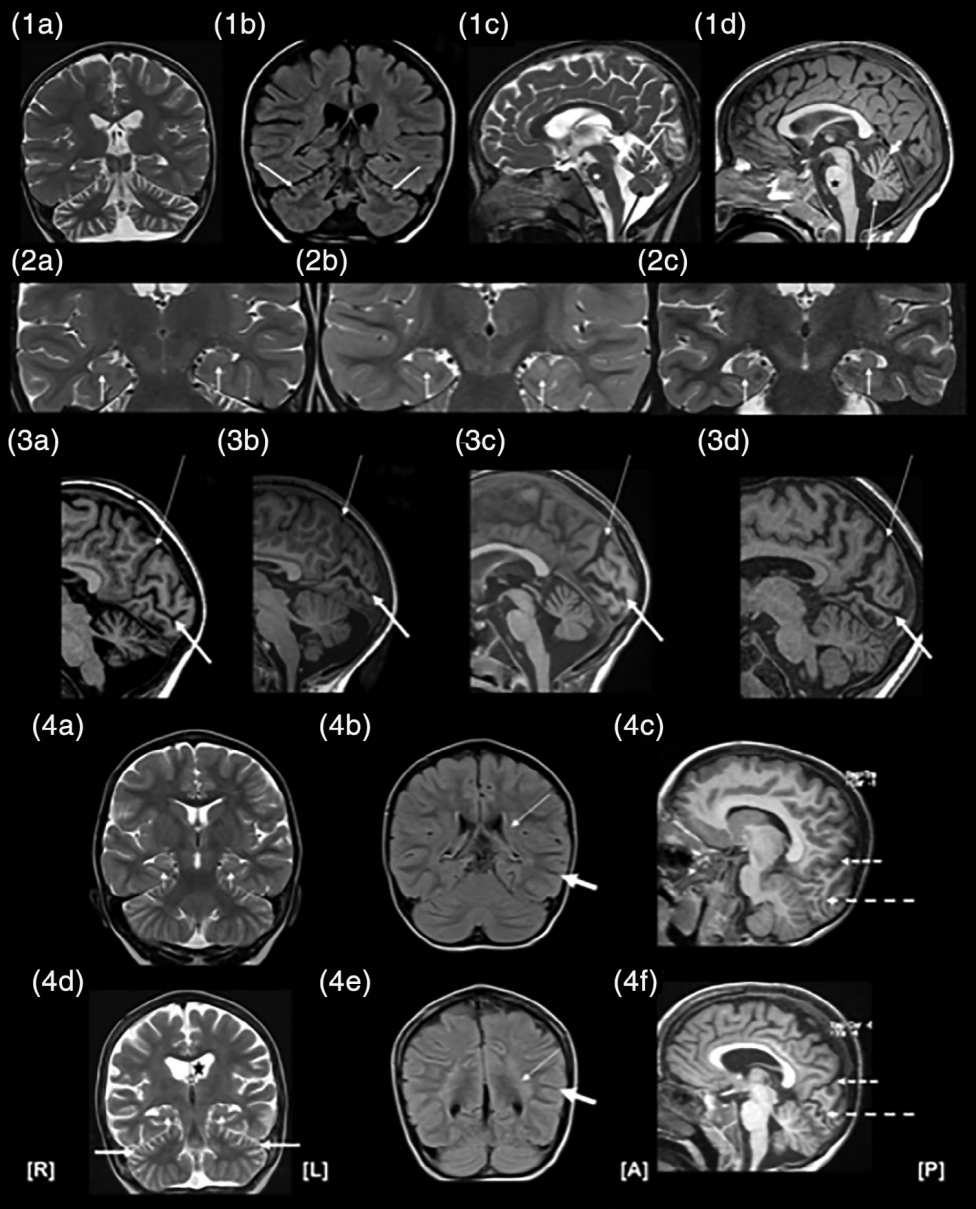
(1a–d) Cerebellar atrophy on magnetic resonance imaging (MRI): (1a) Coronal T2 image at 3 years, 2 months, demonstrating moderately severe generalised cerebellar atrophy. (1b) Coronal fluidattenuated inversion recovery (FLAIR) image at 4 years, 10 months, demonstrating a gradient of cerebellar atrophy from superior to inferior regions. There is also high FLAIR signal in the superior folia (white arrows). (1c) Sagittal T2 image at 3 years, 4 months, demonstrating a gradient of vermian atrophy from superior (white arrow) to inferior (black arrow) regions. Flat pontine belly (star) and normal corpus callosum are also visible. (1d) Sagittal T1 image at 3 years, 7 months, demonstrating a gradient of vermian atrophy from superior (short, thick arrow) to inferior (long, thin arrow) regions. A flattened pontine belly (star) and normal corpus callosum are present. (2a–c) Hippocampal architecture: Coronal T2‐weighted images of three subjects showing reduced internal architecture and mild T2 hyperintensity of the hippocampi. (3a–d) Sagittal MRI patterns of cortical atrophy: Thin arrows indicate the parietooccipital fissure; thick arrows show the calcarine fissure. (3a) Mild parieto–occipital atrophy and moderate atrophy of the calcarine fissure at 3 years, 3 months (note severe cerebellar atrophy). (3b) No significant atrophy of cortical lining of the parieto–occipital sulcus, but severe atrophy of the cortical lining of the calcarine sulcus at 3 years, 3 months. (3c) Severe atrophy of cortical lining of parieto–occipital and calcarine fissure. (3d) Severe, generalised atrophy at 5 years, 7 months. (4a–f) Progressive MRI changes over 1 year, 3 months. Top images (4a–c) at 3 years, 4 months of age, while bottom images (4d–f) are of same subject at age 4 years, 7 months. (4a) Coronal T2 image demonstrating normal ventricles and extraaxial cerebrospinal fluid spaces. Normal‐sized hippocampi with loss of internal architecture (short arrows). (4b) Coronal FLAIR image showing increased signal in the periventricular white matter (thin arrow), with preservation of myelin signal in the subcortical white matter (thick arrow). (4c) Sagittal T1‐weighted image showing normal occipital sulcal spaces. Parieto–occipital fissure (short, dashed arrow) and calcarine sulcus (long, dashed arrow) are indicated for orientation. (4d) Coronal T2 image showing generalised atrophy with ex vacuo dilatation of the ventricles (star) and extra‐axial fluid spaces. Hippocampal volumes are reduced (small arrows), with no visible internal architecture. Superior cerebellar atrophy is also present (thick arrows). (4e) Coronal FLAIR image showing florid periventricular and deep lobar white matter hyperintensity, with preserved signal in subcortical U‐fibres. (4f) Sagittal T1‐weighted image demonstrating marked occipital atrophy with increased sulcal size and cortical bank thinning. Parieto–occipital fissure (short, dashed arrow) and calcarine sulcus (long, dashed arrow) are also shown.

Thirteen patients with CLN2 disease, including two pairs of siblings, were identified. One pre‐symptomatic patient was included in the cohort and was identified at this stage due to a family history of CLN2 disease. Diagnoses were confirmed using enzymatic (*n* = 12) and molecular (*n* = 13) methods. Tables describe the phenotypic features, epilepsy syndromes, diagnostic results (Table 1) and MRI findings (Table 2; Fig. 2). Case reports are presented as Appendix S1 (Supporting Information). One case is described in detail to highlight the early presenting signs and symptoms of CLN2 disease diagnosis, and 12 cases are summarised.

**Table 1:** Clinical characteristics and key presenting features

Pt	Age at study; sex (M/F)	Age at symptom onset	Age at diagnosis	Time to diagnosis	Age at death	Prior language delay	Initial seizure type (underlined), other seizure types	Epilepsy syndrome (initial; evolution)	Other features	TPP1 activity (nmol/h/mg protein; reference range 0.8–2.0)	*CLN2* gene mutation(s)†
1	6 years, 2 months; F	3 years, 3 months	4 years	9 months	—	Yes	Focal to bilateral tonic–clonic	Focal epilepsy	Unsteady on carbamazepine	<0.1	Homozygous c.509‐1G>C
2	7 years; F	2 years, 6 months	3 years, 9 months	1 year, 3 months	—	Yes	Febrile, generalised tonic–clonic, myoclonic, tonic–clonic	Myoclonic‐atonic epilepsy; progressive myoclonic epilepsy	Maculopathy; unsteady on carbamazepine	<0.1	Compound heterozygous (c.1094G>A, p.Cys365Tyr‡; c.1417G>A, p.Gly473Ar‡)
3	Deceased; F	3 years, 1 month	3 years, 10 months	9 months	9 years	Yes	Generalised tonic–clonic, myoclonic	Myoclonic‐atonic epilepsy; progressive myoclonic epilepsy	Ataxia; regression	<0.1	Homozygous c.509‐1G>C
4	Deceased; F	3 years	4 years, 4 months	1 year, 4 months	11 years	No	Focal, focal to bilateral tonic–clonic, myoclonic, atonic	Focal epilepsy; progressive myoclonic epilepsy	Increased seizure frequency with carbamazepine; language regression	<0.1	Homozygous c.509‐1G>C
5	5 years, 2 months; F	3 years, 6 months	4 years, 3 months	9 months	—	Yes	Generalised tonic–clonic	Generalised epilepsy	Atrophy on MRI; ataxia	<0.1	Homozygous c.225A>G‡, p.Gln75=§
6	Deceased; F	2 years, 8 months	3 years, 6 months	10 months	9 years, 9 months	Yes	Absence, myoclonic, focal, generalised tonic–clonic	Atypical absence epilepsy; progressive myoclonic epilepsy	Sister of patient 7	<0.1	Homozygous c.509‐1G>C
7	Deceased; F	3 years, 2 months	1 year, 10 months	Preclinical diagnosis, as affected sibling	10 years, 5 months	No	Absence, generalised tonic–clonic, focal, myoclonic, tonic–clonic	Atypical absence epilepsy; progressive myoclonic epilepsy	Family history of CLN2 disease (sister of patient 6)	Not performed (sibling)	Homozygous c.509‐1G>C
8	5 years; M	3 years, 2 months	3 years, 3 months	1 month	—	Yes	Focal status epilepticus, focal, myoclonic	Focal occipital epilepsy; progressive myoclonic epilepsy	Mild ataxia	<0.1	Homozygous c.509‐1G>C
9	Deceased; M	3 years	3 years, 6 months	6 months	5 years, 6 months	Yes	Generalised tonic–clonic, atonic, myoclonic	Myoclonic‐atonic epilepsy; progressive myoclonic epilepsy	Developmental delay	<0.1	Compound heterozygous (c.622C>T, p.Arg208Ter; c.228C>A, p.Tyr76Ter)
10	Deceased; F	3 years, 6 months	4 years, 9 months	1 year, 3 months	7 years, 11 months	Yes	Focal, myoclonic, generalised tonic–clonic	Focal epilepsy; progressive myoclonic epilepsy	Progressive ataxia; sister of patient 11	<0.1	Homozygous c.509‐1G>C
11	4 years; M	1 year, 6 months	4 months	Preclinical diagnosis, as affected sibling	—	Yes	Language delay, no seizures	NA	Family history of CLN2 disease (brother of patient 10)	<0.1	Homozygous c.509‐1G>C
12	6 years, 6 months; F	3 years	3 years, 4 months	4 months	—	No	Atypical absence, focal, generalised tonic–clonic	Lennox–Gastaut syndrome; progressive myoclonic epilepsy	Language and motor regression; progressive ataxia; family history of CLN2 disease	<0.1	Compound heterozygous (c.622C>T, p.Arg208Ter; c.357dupT, p.Leu120Serfs*18)
13	Deceased; F	2 years, 11 months	4 years, 9 months	1 year, 10 months	7 years, 2 months	Yes		Myoclonic‐atonic epilepsy; progressive myoclonic epilepsy	Ataxia	<0.1	Compound heterozygous (c.509‐1G>C; c.622C>T, p.Arg208Ter)

† Mutation data stated with respect to transcript *NM_000391.3*.

‡ Previously reported in patients affected by CLN2 disease.^17^, ^18^

§ Proven splice site mutation.^17^

MRI, magnetic resonance imaging; NA, not applicable; TPP1, tripeptidyl peptidase 1.

**Table 2:** Brain magnetic resonance imaging (MRI) data from retrospective analyses

Patient	Age at MRI	Time to MRI†	MRI findings on retrospective analysis							
			Cerebellar atrophy	Brain‐stem atrophy	Ventriculo‐megaly	Hippocampal internal structure	Corpus callosum thinning	White matter changes	Thalamic T2 hypointensity	Cerebral atrophy
1	3 years, 7 months	3 months	++ S > I	Yes Pons most affected	+	Normal	Normal	++ P > A	Normal	+
2	3 years, 1 month	7 months	+++	Yes	++	Reduced internal architecture	+ P > A	++ P > A	+	++, +++ P > A
4	5 years, 7 months	2 years, 7 months	++ S > I	Yes	++	Normal	Normal	+	+	++ P > A
5	3 years, 9 months	3 months	++ S > I	Yes	++	Reduced internal architecture	Normal	++	Normal	Normal
6	3 years, 4 months	8 months	+ S > I	Yes Pons	+/++	Normal	Normal	+	+	Normal
8	3 years, 3 months	1 month	++/+++	Yes Pons	+	Reduced internal architecture	+	+	Normal	+/++ P > A
9	3 years, 5 months	5 months	+++	Yes Pons most affected	+/++	Reduced internal architecture	+	+	+	++/+++ P > A
10	4 years, 9 months	1 year, 3 months	+ S > I	Yes Pons	++	Reduced internal architecture	+	+	+	++ P > A
11	1 year, 10 months	NA‡	Normal	No	+	Reduced internal architecture	+	+	Normal	+ P > A
	3 years, 3 months	NA‡	++ S > I	No	+/++	Reduced internal architecture	+	++	Normal	++ P > A
13	3 years, 4 months	5 months	Normal	Yes Pons	+	Reduced internal architecture	Normal	+ P > A	Normal	Normal
	4 years, 7 months	1 year, 8 months	+ Vermis	Yes Pons	+	Reduced internal architecture	+ P > A	+/++ P > A	Normal	++ P > A

† Time to MRI is time from initial seizure to MRI being performed.

‡ Patient diagnosed before symptom presentation.

+, mild; ++, moderate; +++, severe; NA, not applicable; P > A, posterior more than anterior; S > I, superior more than inferior.

### Cohort analyses

Median age at symptom onset was 3 years (range: 1 year, 6 months to 3 years, 6 months), with presenting features including seizures (*n* = 12), known family history of CLN2 disease (*n* = 3) and language delay (*n* = 11). Median age at diagnosis was 3 years, 9 months (range: 4 months to 4 years, 9 months) with a median time to diagnosis of 9 months from symptomatic presentation. Seven patients had died at the time of data collection, at a median age of 9 years (range: 5 years, 6 months to 11 years). All seven patients had end‐stage neurological deficits before their death.

### Epilepsy

Twelve of the 13 patients (92%) had seizures. Median age of seizure onset was 3 years (range: 2 years, 6 months to 3 years, 6 months). Presenting seizure types included focal (*n* = 4), generalised tonic–clonic (*n* = 3) and absence (*n* = 3). During disease progression, multiple seizure types were observed (*n* = 11), including generalised tonic–clonic (*n* = 10), myoclonic (*n* = 9), focal (*n* = 8), atypical absence (*n* = 5), atonic (*n* = 4), gelastic (*n* = 1) and non‐convulsive status epilepticus (*n* = 1).

Multiple EEGs were performed for each patient. A PPR during low‐frequency photic stimulation was noted in eight patients, although this was not always present on the initial EEG. One patient (patient 8) also had a right occipital seizure provoked at 16 Hz. IPS was not performed in two patients. Slow background activity and/or disruption of the background architecture was noted in all patients except the pre‐symptomatic patient (patient 11). Generalised (*n* = 8), focal (*n* = 6) and multifocal epileptiform discharges (*n* = 8) were common, often with a posterior predominance.

On the basis of presenting symptoms, epilepsy syndrome presentation fell into two main groups. Five patients presented with generalised seizures of multiple types, often presenting in an explosive manner and consistent with the syndrome of myoclonic–atonic epilepsy. By contrast, four patients presented with focal epilepsy, with occipital seizures and photosensitivity in one (patient 8) and hemi–clonic seizures evolving to generalised tonic–clonic seizures in another (patient 1). Progressive disorders were suspected in both groups when there was minimal response to standard antiepileptic therapies, such as sodium valproate or the ketogenic diet, particularly for those with myoclonic‐atonic epilepsy and with further regression.

Patients were drug‐resistant, having trialled a median of five antiepileptic treatments (including therapies such as the ketogenic diet), unless they received cerliponase alfa. Common medications included sodium valproate, lamotrigine, levetiracetam, clobazam and clonazepam. Carbamazepine increased ataxia or unsteadiness (*n* = 4).

Three patients are currently receiving cerliponase alfa (patients 1, 8 and 11). None have had generalised tonic–clonic seizures for over 1 year since treatment initiation, with only rare myoclonus or focal seizures in one patient. These were young patients (under 6 years of age), with less exposure to antiepileptic drugs (median: three medications).

### Molecular and enzymatic diagnosis

Diagnosis in CLN2 patients was performed using a combination of enzymology and gene testing. Many older patients within the cohort were diagnosed initially using enzymology followed by gene sequencing, whilst those more recently diagnosed utilised gene panels and whole exome sequencing followed by confirmatory enzymology.

TPP1 enzyme activity testing showed significantly reduced levels (*n* = 12) for all patients, except patient 7, who was the younger sibling of an affected patient and was diagnosed by molecular testing only.

All patients had molecular testing of the *CLN2* gene (Table 1). The most common mutation was c.509‐1G>C, present as a homozygous mutation in eight patients and in combination with c.622C>T (p.Arg208Ter) in one patient. A further two patients were compound heterozygous for the c.622C>T mutation in combination with c.228C>A (p.Tyr76Ter) or c.357dupT (p.Leu120Serfs*18). The age of onset did not differ based on genotype (3 years, 1 month for patients carrying only c.509‐1G>C and/or c.622C>T versus 3 years for patients with one or two other alleles). This was consistent with previous studies (e.g. DEM‐CHILD registry).^8^

### Brain MRI

Brain MRI (1.5–3 Tesla) studies were available for 10 patients, with two patients having follow‐up MRIs available for review (Tables 1 and 2; Appendix S1, Supporting Information; examples of mild to severe changes in Fig. 2). For the three patients were MRI was not available, two were not able to be accessed (patients 3 and 12, both reported as normal), whilst patient 7 did not have an MRI performed as diagnosis was pre‐clinical (affected sibling).

Median time from seizure onset to first MRI in this cohort was 5 months (range: 1 month to 2 years, 7 months). Of the 10 brain MRI films reviewed, all showed cerebellar atrophy (*n* = 8) and/or cerebellar atrophy (*n* = 7, Fig. 2a), although many had been initially reported as being normal. Nine (*n* = 9) patients had thin brain‐stems, with the pons being the most severely affected structure. Hippocampi were normal in size in all patients, but many showed decreased clarity of internal architecture (*n* = 7; Fig. 2b). Cortical atrophy affected posterior regions (medial occipital lobe and posterior cingulate cortex) more than anterior (*n* = 6; Fig. 2c). Mild or moderate ventriculomegaly was identified in all patients. Five patients also showed thinning of the corpus callosum. White matter changes were noted in posterior periventricular, temporal, parietal and occipital regions. In more severe cases, the posterior frontal lobes were also affected. A gradient was present from more severely affected posterior structures to mildly affected anterior structures. No basal ganglia changes were reported, but five patients had a low T2 signal in the thalamus. Although the severity of MRI changes did not correlate with time from seizure onset or age, for individual patients there was an increase in cerebellar and cerebral atrophy, ventriculomegaly and white matter changes on their second MRI (Fig. 2d).

One patient (patient 11) had an MRI prior to symptom presentation. Although cerebellar atrophy was not identified, mild cerebral atrophy was noted with changes in the pons, ventricles, hippocampi and white matter.

## Discussion

We report the phenotypic evolution, MRI features and investigation findings in a cohort of 13 Australian patients with CLN2 disease. Compared with reported cases (DEM‐CHILD registry), our cohort had an earlier median age of diagnosis (3 years, 9 months vs. 4 years, 6 months) and a shorter median time to diagnosis (9 months vs. 1 year, 11 months).^8^ These differences likely reflect increasing disease awareness and improved access to diagnostic testing. Key points arising from our study include epilepsy syndrome recognition in early CLN2 disease, delineation of the MRI features and the improved seizure control within a small subgroup on ERT.

Twelve patients (92.3%) had seizures at presentation, which is more frequent than in the DEM‐CHILD registry (70%).^8^ One third of patients had a focal epilepsy at onset, which has been rarely highlighted in CLN2 disease. Another third presented with myoclonic atonic epilepsy prior to evolution to a progressive myoclonic epilepsy.

Conversely, children on ERT had an improvement noted in markers of epilepsy severity, including long seizure‐free periods. A lower number of antiepileptic drugs had been used in patients on ERT, compared to the remainder of the cohort. This is a remarkable difference to the children in the remainder of the cohort and those in natural history studies.^8^

Expert reanalysis of MRIs in our patients revealed novel findings which may help to clarify the imaging pattern for the early diagnosis of CLN2 disease. Despite previous reports of normal scans, re‐analysis showed cerebellar or cerebral atrophy in all patients at diagnosis, with atrophy observed even prior to seizure onset in one sibling (patient 11). Progressive atrophy was observed in patients with follow‐up MRIs, as seen in previous studies.

We also identified novel, subtle changes in both hippocampal architecture and brain‐stem thinning, particularly affecting the pons. To our knowledge, these changes have not been previously reported. Hippocampal architecture is hypothesised to be altered through neuronal loss in the hippocampal subfields due to frequent or prolonged seizures, or neuronal apoptosis which occurs in CLN2 disease. Brain‐stem thinning may be secondary to progressive atrophy of the cerebrum and cerebellum with loss of projection fibres through the brain‐stem. MRI is often performed early in children presenting with seizures, such that heightened recognition of imaging patterns in early CLN2 disease is critical for early disease detection. This expertise led to a rapid diagnosis in patient 8 just 1 month after his first seizure, rendering him eligible for cerliponase alfa therapy.

A PPR at low‐frequency (1–3 Hz) IPS is a critical pointer to the diagnosis of CLN2 disease. PPR was detected in eight patients (62%), lower than the 93% of patients reported by Specchio *et al*. and 78% reported by Albert *et al*. during initial EEG.^10^, ^12^ We suspect this difference may be explained by variable implementation of IPS, particularly the lack of universal application of lower IPS frequencies in routine paediatric EEG testing. Implementation of IPS, including IPS at slow flash frequencies as part of a standard EEG may aid early diagnosis of CLN2 patients.

## Conclusions

In conclusion, our cohort illustrates the key presenting features and phenotypic evolution of CLN2 disease, including refractory epilepsy occurring on a background of early language delay. CLN2 disease is an ultra‐rare disorder and this case series provides further phenotypic data. We highlight that early MRI brain studies show subtle atrophy in the cerebellum and posterior cortical regions, and also changes in projection regions not previously described (brain‐stem). These findings are an important clue to early diagnostic testing, allowing timely instigation of ERT.

## Supporting information


**Appendix** **S1**. Supporting information.
