# Supplementary material for: Neuronal ceroid lipofuscinosis type 2: an Australian case series
Source: J Paediatr Child Health. 2020 Apr 24;56(8):1210–8. doi: 10.1111/jpc.14890 (PMC7497200; doi:10.1111/jpc.14890)
Supplement: Supplementary file 1 — Appendix S1. Supporting information. [file JPC-56-1210-s001.docx]

# Supplementary materials

## Patient 1: Detailed case description

Patient 1 presented at the age of three years and three months, with focal seizures evolving to prolonged generalised tonic-clonic seizures. The patient was initially treated with carbamazepine and clobazam. Language delay had been noted prior to the onset of seizures, with delays in combining words and articulation difficulties. At the onset of seizures there was a regression of expressive language skills, increasing unsteadiness and falls. These changes were initially attributed to the seizures and anti-epileptic medication, and improved after transition to sodium valproate. At 4 years, she was able to run and ride a scooter, and continued to learn new words and phrases. She developed mildly increased tone at the ankles at the age of four years and some ataxia was noted over the next six months.

EEG at 3 years and 6 months showed bilateral posterior focal epileptiform activity. EEG at 3 years and 8 months showed multifocal and generalised discharges with a slowing of background activity. PPR was reported with IPS at 1 Hz and 15 Hz during both EEGs. Brain MRI at 3 years and 7 months was initially reported as normal, but on review showed cerebellar atrophy. Ophthalmologic examination was normal.

Reduced activity of TPP1 was detected on lysosomal enzyme analysis (<0.1 nmol/h/mg/protein, normal range 0.8-2.0 nmol/h/mg/protein) and molecular analysis of the *CLN2* gene demonstrated homozygosity for the c.509-1G>C mutation at 3 years and 8 months, 5 months after the onset of seizures.

Patient 1 was enrolled into a multicentre trial of intra-cerebroventricular (ICV) ERT with cerliponase alfa (BMN 190-203, a safety tolerability and efficacy study of intracerebroventricular BMN 190 in paediatric patients < 18 years of age with CLN2 disease), at four years and six months of age. There was decline in her gait prior to starting therapy. At six years of age she continues treatment with ERT, which has been well-tolerated without adverse events. She requires assistance with walking and continues to learn new words. She doesn’t require feeding support and has a normal sleep pattern. She continues treatment with sodium valproate and clobazam and has been seizure-free for over 18 months, since starting ERT.

### Patient 2

Although patient 2 presented with seizures, language deficits were also identified prior to diagnosis. The patient became unsteady whilst walking, and had difficulty using stairs. Early pseudo-focality on EEG led to treatment with carbamazepine, which made her more unsteady. Maculopathy was present at an early stage. Generalised tonic-clonic seizures and myoclonus were treated with sodium valproate, clobazam, lamotrigine and levetiracetam. Dystonia was present, with ongoing dystonia at times when patient 2 was febrile and unwell. Constipation also seemed to trigger further dystonic episodes, and gabapentin and clonidine were initiated. Dystonic eye movements (prolonged upgaze) were also observed. Feeding support through percutaneous endoscopic gastrostomy (PEG) was required. Sleep difficulties were treated with melatonin, clonidine and chloral PRN. The patient, now aged 6 years, is currently in the end stages of CLN2 disease with no language ability nor voluntary motor function. She has ongoing myoclonus and has minimal visual interaction. The palliative care team have been involved from soon after diagnosis.

### Patient 3

Mild concerns over language development were present before the development of seizures, and at diagnosis the patient also had difficulty walking. As the disease progressed, the patient also developed ataxia, myoclonus and behavioural problems. A range of anti-epileptic drugs (AEDs) – sodium valproate, levetiracetam, clobazam, topiramate and phenobarbitone – were used to manage seizures, which included generalised tonic-clonic seizures, atypical absences, atonic seizures, myoclonic jerks, and gelastic seizures. A ketogenic diet was found to be effective in reducing drop and absence seizures, although this effect was not maintained. Feeding support via PEG, and melatonin and behavioural management for sleep problems were also required. She was unable to mobilise and had lost language, with difficult behaviour at times. An end of life plan was in place, and the patient died at age 9, in end stage disease.

### Patient 4

Language deficits were reported 6 months after the development of seizures, and at diagnosis the patient experienced progressive myoclonic, atonic and tonic seizures, and declining motor function and ataxia. Seizures were treated with carbamazepine, sodium valproate, clobazam, levetiracetam and topiramate. Carbamazepine was associated with increased seizures, irritability, staggering and vagueness, while thrombocytopenia developed when the patient was treated with sodium valproate, and feeding decreased while using topiramate. Carbamazepine was withdrawn and sodium valproate dose decreased following these events. The patient was fed via PEG, and also required behavioural management to reduce the risk of sleep difficulties. The patient experienced sustained periods of distress, thought to be secondary to discomfort, in the end stages of disease, and died at the age of 11 years. An end-of-life plan was in place and the patient received palliative care.

### Patient 5

Patient 5 was diagnosed by exome testing after presenting with generalised tonic-clonic seizures. Although ataxia and subtle language deficits were present initially, the patient still showed minor motor and language deficits at 4 years, 6 months of age, and seizures were infrequent. Seizures were generalised and treated with levetiracetam and sodium valproate. No support was required for feeding or sleeping issues. At this stage, her family have elected not to proceed with ERT.

### Patient 6

At diagnosis, patient 6 presented with significant language and developmental delays, and was also unsteady and ataxic. Expressive language was more severely affected than receptive language. Vision deteriorated as the disease progressed, and motor and language functions continued to decline. Seizures were managed using topiramate, levetiracetam, sodium valproate, lamotrigine, and clonazepam. The patient also required feeding support and was prescribed omeprazole to manage gastrointestinal issues. As the disease progressed the patient received allied health therapy, and required a wheelchair. The patient died at the age of 9 years.

### Patient 7

This patient was diagnosed through genetic testing before symptoms presented, as she was the younger sister of patient 6. When seizures developed, levetiracetam, topiramate and clobazam were prescribed. Spasticity, ataxia and stagnation of language skills developed as the disease progressed, and a nasogastric feeding tube was also required. The patient did experience some fluctuating sleep difficulties, but overall sleeping was considered to be good. The rate of deterioration accelerated from the age of 4 years, and the patient died aged 8 years.

### Patient 8

Patient 8 presented at 3 years 2 months with focal status epilepticus on a background of mild speech delay. He had acquired single words at 18 months and had a history of persistent sleep problems. He had frequent focal impaired awareness seizures with pallor, oral automatisms, head and eye deviation, lasting 3-12 minutes. EEG showed right temporal and left occipital discharges. He was prescribed carbamazepine, levetiracetam, and sodium valproate for seizures. He developed mild ataxia. Myoclonic seizures commenced at 3 years 8 months.

He was commenced on cerliponase alfa at 3 years 11 months through a compassionate access scheme. His seizures settled at 4 years 1 month. He has had no complications associated with therapy, other than low-grade fever, for which he receives pre-infusion antihistamine and paracetamol. His receives physiotherapy, occupational therapy, and speech therapy. He is mildly ataxic but can walk run without support.

### Patient 9

Patient 9 had language delay and an unsteady gait at seizure onset. He was initially admitted to hospital under the care of the cardiology team to exclude a cardiac cause, as he was experiencing atonic seizures. His language skills regressed, and ataxia increased in severity. His seizures were managed with sodium valproate, clonazepam, clobazam, levetiracetam, and ethosuximide. Sedative effects occurred on higher doses of clonazepam, predisposing him to upper respiratory tract infections. Patient 9 also had difficulty sleeping from an early disease stage and could behave aggressively. He developed markedly increased palatal and limb myoclonus after gastrostomy placement and died at age 5 years.

### Patient 10

Patient 10 had language delay prior to diagnosis, and by the time of diagnosis was experiencing worsening ataxia and increased seizure frequency. She had regression of motor and language, more severe than expected due to her epilepsy. Seizures were managed using sodium valproate, clobazam, clonazepam, levetiracetam, and phenytoin, and midazolam and phenobarbitone at end stage disease. Clonazepam increased salivation and swallowing difficulties, and phenytoin was associated with increased severity of ataxia. Dystonia was difficult to manage. PEG was required to support feeding, and the patient had sleep disturbances from an early disease stage, requiring treatment with melatonin. The patient died at the age of 7 years, while receiving palliative care.

### Patient 11

The younger brother of Patient 10, the mother was pregnant when the older sibling was diagnosed, and so this patient was diagnosed before symptoms developed. Language delay was present from 1 year and 6 months. The patient is now aged 3 years, and receives melatonin for sleep disturbance. EEG abnormalities have been associated with brief vacant spells, and although no definite seizures have been reported, the patient is treated with sodium valproate. He is receiving compassionate treatment with cerliponase alfa.

### Patient 12

A second cousin of a patient with late-infantile NCL, CLN2 disease was considered at presentation of symptoms. The patient is now aged 5 years, and is only able to move by crawling, and has minimal language abilities. The patient has experienced progressive truncal and gait ataxia, spasticity (treated with botulinum toxin), hypotonia and myoclonus (treated with levetiracetam), which have become more prominent and severe with disease progression. Likewise, language has regressed with the disease, and the patient now only occasionally produces recognisable single words. She has atypical absence, focal motor, and generalised seizures that are responsive to sodium valproate and clobazam. Sodium valproate treatment resulted in drowsiness, which was resolved by decreasing the dose. The patient had severe oropharyngeal dysphagia and is fed via PEG support. She also experienced multiple nocturnal arousals and receives melatonin for sleep initiation. Risperidone has been prescribed for neurobehavioural lability. She is now managed through a coordinated team, including palliative, metabolic, neurological, rehabilitation, and general paediatric support, and is awaiting a decision on the compassionate use of cerliponase alfa for late-stage disease.

### Patient 13

Patient 13 presented at age 2 years and 11 months with the epilepsy syndrome of myoclonic-atonic epilepsy, with generalised tonic-clonic, myoclonic, atonic and absence seizures; she previously had febrile seizures. She had pre-existing language delay and delay in fine motor skills. Her EEG showed generalised spike-wave activity and she was commenced on the ketogenic diet. Her seizures were resistant to many anti-epileptic drugs: valproate, lamotrigine, ethosuximide, clobazam, midazolam, topiramate, levetiracetam, diazepam and lacosamide. She then showed profound developmental regression, with myoclonus, tremor, ataxia, dystonia and spasticity. She developed a progressive myoclonic epilepsy, and eventually became wheelchair-bound and non-verbal. Baclofen relieved spasticity to some extent, and orthotics were used. PEG feeding was implemented at age 5 years. Sleep disturbance was managed with melatonin. The patient had nystagmus, scoliosis, and dementia, and was prescribed domperidone for nausea and glycopyrrolate for excessive salivation. The patient died at the age of 7 years, 2 months.
